# Automated Focal Plane Merging From a Stack of Gonioscopic Photographs Using a Focus-Stacking Algorithm

**DOI:** 10.1167/tvst.11.4.22

**Published:** 2022-04-22

**Authors:** Masato Matsuo, Nana Kozuki, Yuina Inomata, Yoshiki Kumagai, Ryosuke Shiba, Koji Hamaguchi, Masaki Tanito

**Affiliations:** 1 Department of Ophthalmology, Shimane University Faculty of Medicine (Matsuo, Kozuki, Inomata, Tanito), Izumo, Japan; NIDEK CO., LTD., Gamagori, Japan (Kumagai, Shiba, Hamaguchi)

**Keywords:** glaucoma anterior segment, image analysis, automated gonio-photography, focal plane merging, Fourier Burst Accumulation

## Abstract

**Purpose:**

The purpose of this study was to investigate the utility of automated focal plane merging with the collection of gonio-photographs with different depths of field (DOF) using an established focus-stacking algorithm.

**Methods:**

A cross-sectional study was conducted at Shimane University Hospital, Izumo, Japan. Sixteen eyes from 16 subjects from the glaucoma clinic were included in this study. Image processing was performed for the images of 16 eyes from 16 angle sector following the successful gonio-photography. The 256 sets of focus-stacked and best-focused images were prepared in random order and were compared for the DOF and informativeness to diagnose angle pathology by masked observers in each set as the subjective assessments. Moreover, the energy of the Laplacian (average |Δ*I*|), which is an indicator of image sharpness between the photographs with and without the focus-stacking processing was also analyzed with the Laplacian filter as the objective assessment.

**Results:**

The automated image processing was successfully performed in all stacks of images. The significant deepening of DOF and improvement of informativeness achieved in 255 (99.6%) and 216 (84.4%) images (*P* < 0.0001 for both, sign test) and the energy of the Laplacian also significantly increased in 243 (94.9%) images (*P* < 0.0001, sign test).

**Conclusions:**

Focal plane merging by the automated algorithm can make the gonio-images deeper focus compared with the paired best-focused images subjectively and objectively, which would be useful for angle pathological assessment in clinical practice.

**Translational Relevance:**

Focal plane merging algorithm for the automated gonio-photography can facilitate the angle assessment by providing informative deep-focus image, which would be useful for glaucoma care.

## Introduction

Glaucoma is the leading cause of irreversible blindness worldwide, with the global number of affected individuals expected to rise to 111.8 million by 2040 as the elderly population grows.^1^^,^^2^ Glaucoma is a progressive neurodegenerative ocular disease that causes visual field loss due to damage of the retinal ganglion cells and can be classified into two broad categories (open or closed angle; primary or secondary) according to the iridocorneal angle assessment.^3^ The angle evaluation is required for glaucoma diagnosis and clinical evaluation because the effective treatment methods and policies differ depending on the glaucoma types.^4^^–^^10^

The current clinical standard for angle evaluation is manual gonioscopy, a contact method developed in the 1800s that requires topical anesthesia and patient cooperation.^6^^,^^7^^,^^9^^,^^11^^,^^12^ Gonioscopy using an indirect gonio-lens commonly used in daily practice offers several advantages: provision of a real-time in vivo image with rich chromatic information; visualization of specific angle structures or additional features of interest with a magnified view; and the ability to perform both static and dynamic gonioscopy (compression or indentation gonioscopy). However, the technique is subjective without high confidence and reproducibility and requires both expert and experienced clinicians.^12^^–^^17^ Moreover, the implementation rate is underperformed even among ophthalmologists.^13^^,^^18^ The lack of highly reproducible, expeditious, and practical methods for recording angle findings or images often makes the manual gonioscopy inappropriate for glaucoma care.

The Gonioscope GS-1 (Nidek Co., Gamagori, Japan) is a gonioscopic camera that covers 360 degrees of the iridocorneal angle and provides true-color images in less than 1 minute per eye similar to static gonioscopy with indirect gonio-lens. The device can preserve series of gonio-photographs with different focus distance in every 16 angle sectors and successively display the best-focused image in each sector, which is focused on the angle recess and selected automatically.^6^^–^^8^ The device can accurately record angle findings using the automatic camera, which would be clinically useful in screening, diagnosis, and long-term follow-up. However, it could not provide a picture with all planes in focus. Even the best-focused image has some blurred and out of focus areas, and the other saved different-focused angle images could be informative in angle analyzes. Furthermore, even if it is an image of the same sector, the gonioscopic images have different blurs at each part of the image.

Focal plane merging is a new imaging technique for creating a single deep-focus image from a stack of images collected with different depths of field (DOF).^19^^,^^20^ The focal length of the gonioscope as defined by optics and photography, is the distance between the center of the optical lens and the focal point, which determines the real image plane that is in focus. Furthermore, the DOF is determined by the distance from the nearest object plane in acceptable focus to that of the farthest plane also simultaneously in focus. With the focus-stacking image-editing technique using the Fourier Burst Accumulation (FBA), multiple images of varying focal lengths can be spliced together to produce improved quality and accuracy of imaging.^19^^,^^20^ In this study, we developed a novel focus-stacking algorithm (Nidek Co., Gamagori, Japan) that correlates with various blurs for each part in one GS-1 image by applying FBA for each local region and finally synthesizing, which needed to be validated for usefulness in clinical settings. Therefore, our goal was to explore the capabilities of the novel technique and to investigate the utility of the automated focal plane merging with the collection of gonio-photographs with different DOFs using the established algorithm in various scenarios typically encountered in a glaucoma subspecialty clinic.

## Methods

### Participants

A cross-sectional clinic-based study was conducted at Shimane University Hospital, Izumo, Japan. The institutional review board (IRB) of Shimane University Hospital reviewed and approved the research named “Automated focal plane merging with gonioscopic images using focus-stacking technique” (No. 20200114-1). The Declaration of Helsinki was followed in all research. The IRB approval did not require each patient to provide written informed consent for publication; instead, the study protocol was posted at the study institutions to notify participants about the study, and they were allowed to opt-out from the research. In April 2021, the studies included 16 eyes of 16 individuals who had a successful gonio-photography with GS-1 at Shimane University Hospital for specialized glaucoma care and performed automated focal plane merging. If both eyes met the eligibility criteria, the patient information was blinded and only one eye was randomly selected for analysis. All patients were Japanese.

### Gonioscope GS-1 Imaging

The Gonioscope GS-1 is an anterior-segment imaging device that generates true-color gonio-images automatically and in a standardized manner. It covers 360 degrees of the angle. According to our previous reports,^6^^,^^21^ after applying topical anesthetic eye drops and using gel coupling, gonio-images of the entire circumference were captured by GS-1 with the participants fixating in primary gaze in a darkened room as during a typical examination. A prism with 16 mirrored facets, each covering about 30 degrees, was used to detect the angles, and a series of 16 images were taken as if performing an indirect gonioscopy with a Goldmann lens. The instrument then automatically captured and saved the images (1280 × 960 pixels, 96 × 96 dpi) with the best focus within the sequential 15 depths of focus for each position. Finally, we gathered 240 gonio-images in each of 16 sectors with 15 focal planes (16 × 15) for each eye.

### Automated Focal Plane Merging

**Figure 1.**
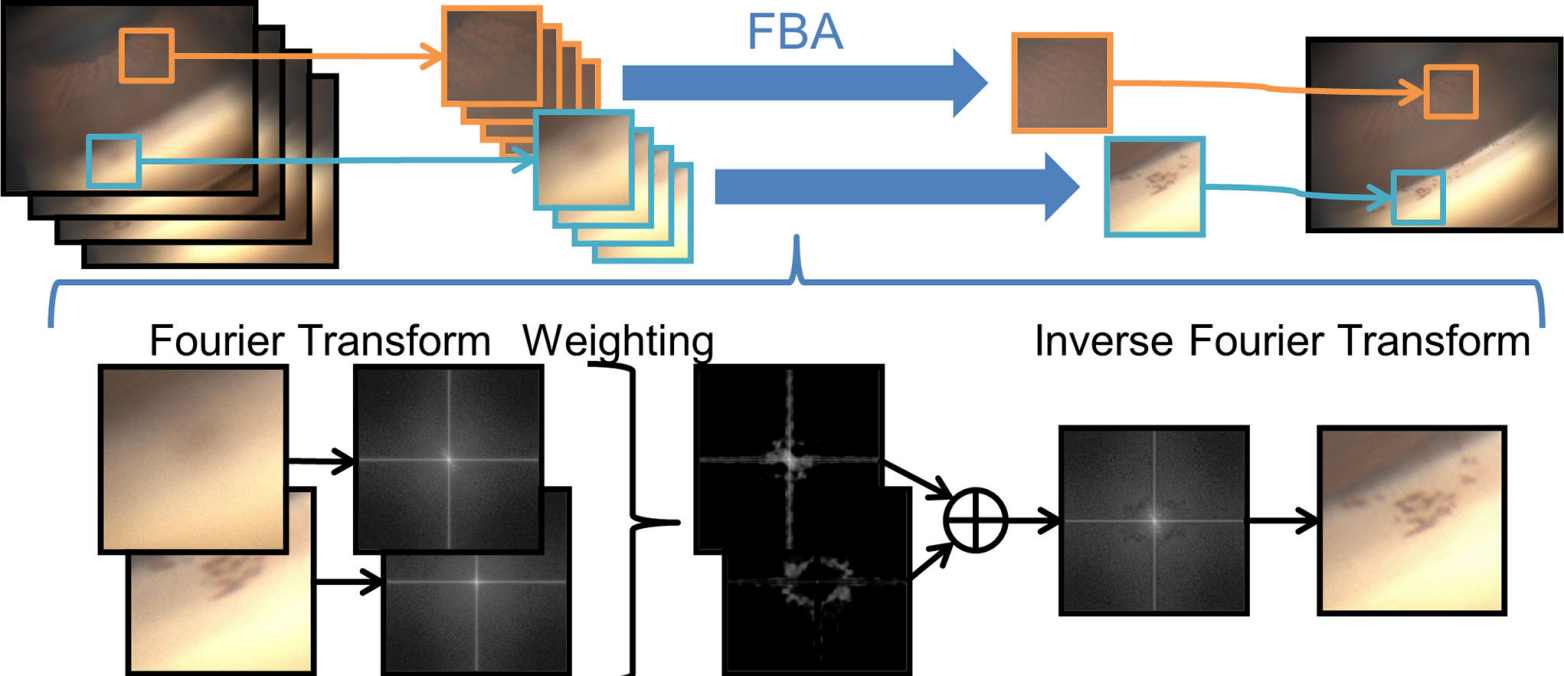
The overview of the focus-stacking algorithm to create a single deep-focus gonio-image with improved quality and accuracy from a stack of images collected with different depths of field. The method corresponds to different blurs for each part in one gonio-image by applying Fourier Burst Accumulation (FBA) for each local region.

The established automated focal plane merging algorithm is based on the FBA technique. The image-editing technique of the FBA is a useful method for removing image blur that is uniform over the entire image, such as camera shake, it is insufficient for a focal plane merging of gonioscopic angle images with different blurs for each position in one image.^19^ The FBA technique can be applied to the GS-1 image because the blur can be regarded as uniform in the local area. Thus, to correspond to different blurs for each part in the image, we developed the novel technique applying the FBA for each local region (256 × 256 pixels) separately, which is shifted by 128 pixels, and finally synthesized them as shown in Figure 1. In this algorithm, seven consecutive images in the foreground including the best-focused image were used. It conducts the Fourier transforms and a weighted average in the Fourier domain, with weights depending on the Fourier spectrum magnitude, for each sector of the given seven images based on the best-focused image, and then, the inverse Fourier transform is done for each part of the image. The images of those parts are finally combined into one focus-stacked image (1280 × 960 pixels, 96 × 96 dpi), which can be synthesized without explicitly solving any blur estimation and subsequent inverse problem the same as the original FBA algorithm. Using the stack of more than seven images at varying focal lengths during the focus-stacking procedure may help improve overall sharpness; however, we found excellent results when limiting to seven total pictures around the best-focused one. The image processing took about 1 minute per eye on windows 10 operating system using Intel Core i3-7020U central processing unit with 4 gigabytes of random access memory. Finally, 256 focus-stacked images of 16 eyes were obtained in each of 16 sectors (16 × 16) for analysis.

### Subjective Comparisons Between Focus-Stacked and Best-Focused Images

The 256 best-focused images of 16 eyes were automatically obtained in each of 16 sectors (16 × 16) during the initial image acquisition with the gonio-photography. After blinding the patient information, an author (N.K.) prepared 256 sets of focus-stacked and best-focused images in a random order, and a masked observer (M.T., a glaucoma specialist with >20 years of experience) in each set compared the DOF and informativeness. In the first test, the distance from the nearest object plane in acceptable focus and the farthest plane in the focus was simultaneously used to determine which of the pair of images had the deeper DOF. Alternatively, it was the comparison of the size of the area in the image where the angle structure was clearly visible. In the second evaluation, it was found which of the two images were more useful in diagnosing angle pathology clinically. The informativeness was determined from the standpoint of a glaucoma specialist because the region of interest in glaucoma care is different for each individual. Particularly, it is selecting an image that provides the clearer and more detailed view in the region of interest in the angle; however, even a blurry image could still provide information for clinical decision making. Subjective assessments for calculating interobserver agreement were performed in a similar manner by the other masked observer (author M.M.). As a result, the concordance rates were 99.6% (deepness of DOF) and 94.5% (informativeness to diagnose angle pathology), respectively.

### Objective Comparisons Between Focus-Stacked and Best-Focused Images

**Figure 2.**
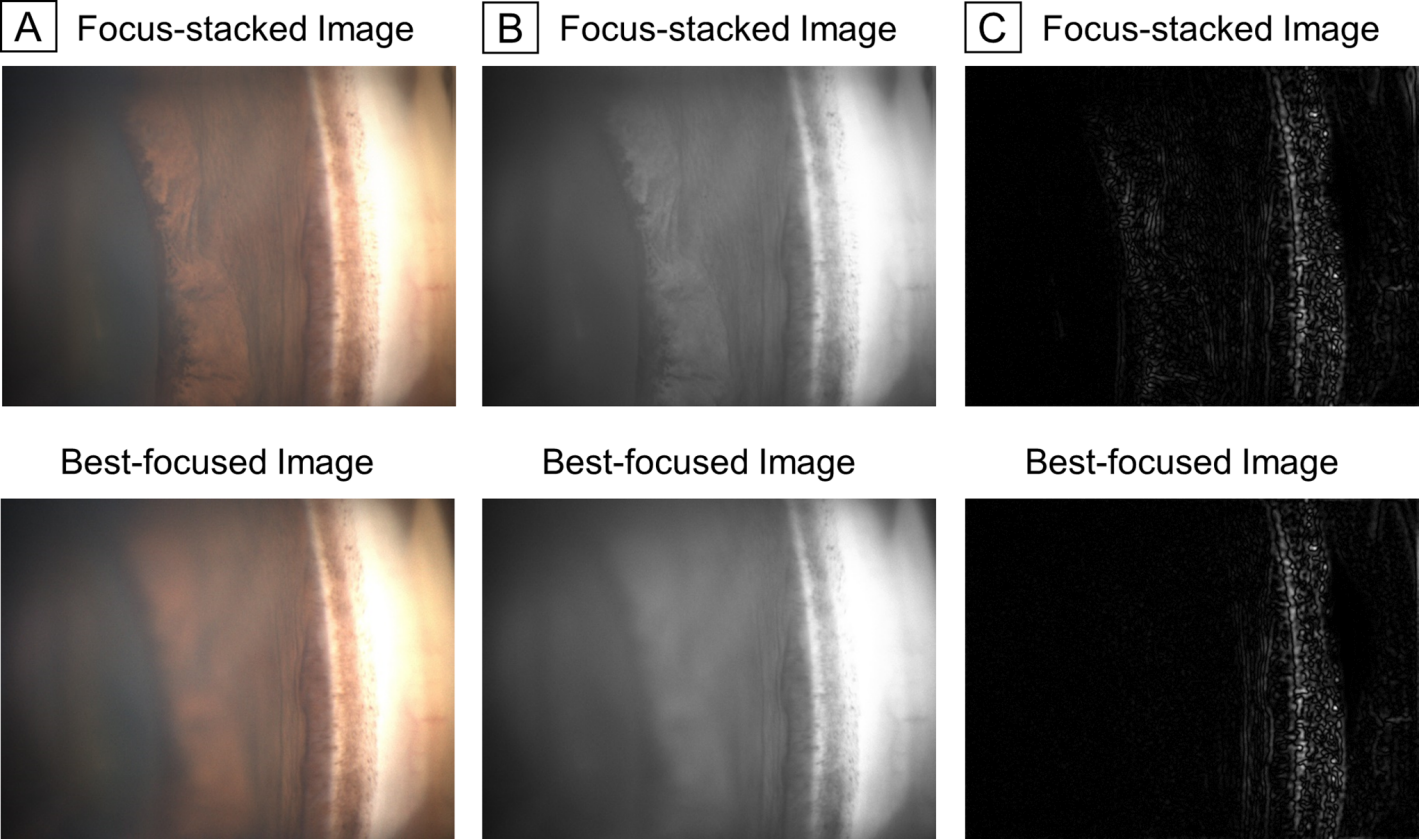
The example of image processing for objectively analyzing the sharpness of an image. (**A**) The pair of original gonio-photographs. (**B**) The pair of **A** images after conversion to grayscale. (**C**) The pair of results after the Laplacian filter processing with **B** image (|Δ*I*|). The focus-stacked image exhibited more edges (*white part*) than the best-focused one visually.

The 256 sets of focus-stacked and best-focused images were also prepared and converted to grayscale. The energy of the Laplacian, which is a measure of image sharpness was analyzed with the Laplacian filter (Nidek Co., Gamagori, Japan) and compared for each set. What is obtained from the filter is the absolute value of the second derivative of the image (|Δ*I*|), which takes a large value in pixels with strong edges. The sum of the second derivative of the entire image is effective in evaluating focus according to the previous report.^22^ Therefore, we defined the energy of the Laplacian as the average absolute value of the second derivative of 1280 × 960 pixels gonio-image (average |Δ*I*|) for focus measure in this study. Thus, the clearer the image, the greater the energy of the Laplacian. For example, one of the pairs of original gonio-photographs, those of grayscale images, and those of the results after the Laplacian filter processing (|Δ*I*|) are shown in Figure 2, and the focus-stacked image exhibited the more edges and the larger energy of the Laplacian than the best-focused image. In the analysis, it was determined which of the pair of images had larger energy of the Laplacian.

### Statistical Analysis

JMP Pro version 14.2 statistical software was used for all statistical studies (SAS Institute, Inc., Cary, NC). The sign test was used to compare the assessments for the gonio-photographs with and without the focus-stacking processing in total, for each sector, and each subject. To see if the energy of the Laplacian follows a normal distribution, and the Shapiro–Wilk Normality test was performed. Statistical significance was defined as *P* < 0.05.

## Results

**Figure 3.**
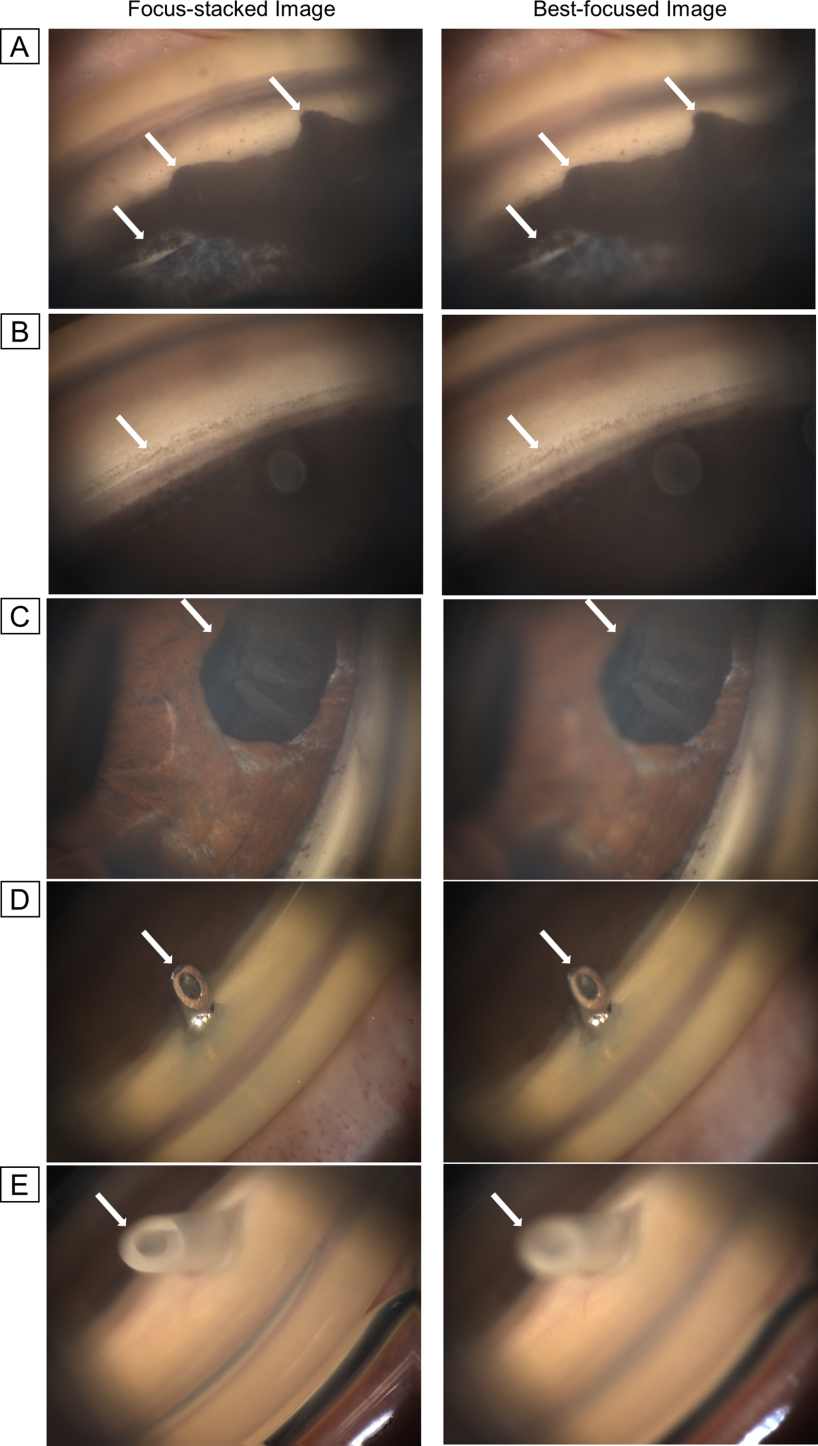
The representative pairs of focus-stacked and best-focused images, in which the focal plane merging technique achieved the significant deepening of the depth of field and improvement of informativeness and increased energy of the Laplacian. The salient areas of interest are indicated by *white arrows*. (**A**) The images show tall tent-shaped peripheral anterior synechiae (PAS). The focus-stacked image has clearer PAS boundaries and more detail about iris damage. (**B**) The pair of images is an example of pseudoexfoliation glaucoma. The shape and shade of angle pigmentation can be observed in the focus-stacked image compared with the best-focused one. (**C**) The pair of images is an example after trabeculectomy. Compared with the best-focused image, the focus-stacked image clearly shows the peripheral iridectomy scar and iris damage. (**D**) As an example, after the EX-PRESS Glaucoma Filtration Device (Alcon Laboratories, Fort Worth, TX) procedure, the DOF in the focus-stacked image is deep enough to be observed the angle from the tip of the device to the insertion area compared with that of the best-focused one. (**E**) The pair of images is an example after the Ahmed glaucoma valve implantation (Model FP-7; New World Medical, Rancho Cucamonga, CA) into the anterior chamber. Compared with the best-focused image, the focus-stacked one has a wider range of focus, and it can be observed well from the tip of the device to the insertion part. Moreover, its lumen can be seen.

All stacks of images were successfully processed using automated image processing in a minute per eye, and 256 sets of focus-stacked and best-focused images were prepared for analysis. Then, subjective and objective comparisons were made between focus-stacked and best-focused images. The Laplacian energy (average |ΔI| ± standard deviation) assessed with the Laplacian filter for the photographs for the objective evaluation was 1.00 ± 0.26 (focus-stacked images), and 0.79 ± 0.19 (best-focused images), and the Shapiro–Wilk statistic was 0.975 in focus-stacked images (*P* < 0.01) and 0.916 in best-focused images (*P* < 0.01). Therefore, we considered that the energy of the Laplacian would follow a nonparametric distribution and selected to use the sign test to analyze for consistent differences between the pairs of observations for each subject. Moreover, we did not compare the subjective interpretation with the objective measurement, which could be helpful for implementing interpretability scores like signal strength in optical coherence tomography. The Table demonstrates the primary outcomes measured. Focus-stacked images had greater proportions of images with deeper DOF (255 vs. 1, *P* < 0.0001, sign test) and more informative images (216 vs. 40, *P* < 0.0001, sign test) compared to best-focused images in subjective assessments by a glaucoma specialist. Moreover, the objective measurement showed that focus-stacked images had a higher proportion of images with larger energy of the Laplacian (243 vs. 13, *P* < 0.0001, sign test) compared to best-focused images. Therefore, the focus-stacked images were significantly superior to the best-focused images in all consideration items. Figure 3 shows the representative pairs of focus-stacked and best-focused images.

**Table.** Comparisons Between the Assessments for the Gonio-Photos With and Without the Focus-Stacking Processing

Assessment	Which was Better, the Focus-Stacked Image or the Best-Focused Image (*n* = 256)	*P* Value
Deepness of DOF, number of focus-stacked images : best-focused images (percentage of focus-stacked images)	255 : 1 (99.6)^*^	<0.0001^a^
Informativeness to diagnose angle pathology, number of focus-stacked images : best-focused images (percentage of focus-stacked images)	216 : 40 (84.4)^*^	<0.0001^a^
Energy of Laplacian, number of focus-stacked images : best-focused images (percentage of Focus-stacked images)	243 : 13 (94.9)	<0.0001^a^

Abbreviation: DOF, depth of focus.

* It was a forced choice method and the observer could not state that both images were comparable.

a Sign test.

We performed additional analyzes independently for each sector and each eye to account for potential bias caused by the same sector or eye. In all of the consideration items for each sector analyzes, focus-stacked images were superior to the best-focused images, the same as on the primary outcomes shown in the Table. Furthermore, significant deepening of the DOF, improvement of informativeness, and increase of energy of the Laplacian was achieved in almost all evaluations. Supplementary Table S1 shows the statistical significance between the focus-stacked and the best-focused images for each sector. However, they also achieved in almost all the evaluation items for each eye as, shown in Supplementary Table S2, however, in the subject “14,” the focus-stacked images were not significantly superior in the informativeness to diagnose angle pathology and in the energy of the Laplacian to the best-focused images. Thus, image processing was not effective subjectively and objectively in the subject “14” images. Supplementary Figure S1 shows the pair of gonio-images in the inferior sector of the subject “14.” To infer when the algorithm would not be useful, we investigated the medical histories of the eyes from which the angle images were taken. Supplementary Table S3 shows the demographics and clinical characteristics of study subjects. The subject “14” was a 76-year-old man who had a history of blunt ocular trauma on his left eye with a baseball ball more than 50 years ago. His left eye was observed, and it was found that the angle recession with widening of the ciliary body band in greater than 180 degrees of the angle and had open angle glaucoma. Thus, the eye had an extreme wide angle, and the captured best-focus image exhibited various in-focused area.

## Discussion

We created a focus-stacking algorithm that corresponds to different blurs for each part of the gonio-image in this study by applying FBA to each local area and then synthesizing them. Then, using a collection of gonio-photographs with various DOF in various patients encountered in a glaucoma section of a tertiary care center, we investigated the capabilities of the recent FBA technique and validated the algorithm's utility.

As shown in the Table and Figure 3, we could demonstrate that the angle image processing achieved not only the deepening of DOF and improved informativeness in the subjective assessments by a glaucoma specialist but also the increased energy of the Laplacian in the objective evaluation with the Laplacian filter in total. In other words, when compared to the best-focused image in general, the focal plane merging algorithm was able to successfully produce clinically enhanced gonio-images with wider DOF using the stack of GS-1 photographs. However, we adopted a forced choice method in the subjective evaluation, and the observer could not state that both images were comparable. Therefore, the choice may have been biased toward clearer images, even if both images are equally useful clinically.

We conducted an additional detailed analysis for the potential bias caused by the same sector or the same eye, as shown in Supplementary Tables S1 and S2. Same as on the primary outcomes shown in the Table, the focus-stacked images were superior to the best-focused images in almost all the consideration items for each sector analyzes with statistical significance. Therefore, there seemed to be little or no bias for different sectors. However, the superiority of the focus-stacked image also achieved in almost all the evaluation items for each eye, however, in the subject “14,” the focus-stacked images were not significantly superior subjectively and objectively. The subject “14” eye with various angle recession had an extreme wide angle, and the captured best-focused image alone exhibited several in-focused areas. Thus, it could be considered that if the distance of angle structure from the GS-1 gonio-prism changed little in the image, the focal plane merging processing would not be necessary, but rather the image could become blurry by overlaying the other images on the best-focused one. Therefore, the automated algorithm might not be useful in a few cases, such as angle recession, where the angle is wide enough. However, to focus on the entire image, slightly overlapping adjacent focal planes are preferred. Because the area in focus is smaller on the camera side compared to the deepest part of the angle, the focus-stacked image can be improved using images taken with a finer focus step forward of the angle. Moreover, because the clinical utility of the focal plane merging algorithm can vary depending on individual's condition, clinical purpose, and region of interest, a different data set may yield different results. The limits of the usefulness of this algorithm will need to be considered for future clinical use.

In the field of ophthalmology, improving image quality must be extremely beneficial.^6^^–^^9^ First, it could improve the glaucoma diagnostic rate and glaucoma treatment results based on objective angle findings in the image. As described earlier, gonioscopy is essential for glaucoma diagnoses and clinical evaluations and documenting angle findings with high reproducibility is mandatory for glaucoma care. Because an angle can be seen at a glance in a single focused-stack image, the oversight of angle findings will be reduced. Additionally, because the enhanced angle images can be analyzed post hoc, physicians can make detailed observations repeatedly, including image manipulation to magnify any abnormalities.^10^^,^^23^ Thus, the algorithm would be helpful for decision making concerning the degree of angle opening, suitability for performing certain procedures, such as laser trabeculoplasty or angle-based surgical procedures. Moreover, the software should facilitate telemedicine or tele-glaucoma care with the focus-stacked images in remote locations, which could improve glaucoma diagnostic rates and reduce preventable visual loss via early detection.^9^^,^^24^ Additionally, it would be useful for scientific and educational purposes. Photographic data used for research and training should maintain high-quality and accurately reflect the source material. However, the need for training to interpret the angle images, the intraobserver and interobserver variability in interpretation, and the lack of dynamism that comes from assessing static images are still a challenge.^9^^,^^25^

In conclusion, we could develop the focus-stacking algorithm that corresponds to different blurs for each part in one GS-1 image by applying FBA for each local region and validated for usefulness in clinical settings for glaucoma care. This study is a proof of focus-stacking technique to render the improved quality of imaging in the field of ophthalmology. Our proof of concept with this method demonstrates markedly improved image quality subjectively and objectively with existing gonio-photographs. However, the automated algorithm might not be useful in a few cases because of the variable angle conditions, clinical purposes, and region of interests. Overall, the algorithm offers a simple and practical way for improving the quality of photographic imaging requiring no additional training, which would promote the application not only to ophthalmology but also to other diagnostic imaging fields. Currently, automatic diagnosis techniques for various clinical images using deep learning algorithms are being developed,^26^ and the improvement of image quality can improve the level of their diagnostic capability.^27^ Therefore, the focus-stack algorithm, which can provide stable images with good quality, is expected to facilitate automatic diagnosis using deep learning.

## Supplementary Material

Supplement 1
