# Supplementary material for: Automated Focal Plane Merging From a Stack of Gonioscopic Photographs Using a Focus-Stacking Algorithm
Source: Transl Vis Sci Technol. 2022 Apr 22;11(4):22. doi: 10.1167/tvst.11.4.22 (PMC9055566; doi:10.1167/tvst.11.4.22)
Supplement: Supplement 1 [file tvst-11-4-22_s001.docx]

**Supplementary** **materials**

**Supplementary Tables**

**Supplementary Table 1.** Statistical significances between the focus-stacked and the best-focused images for each sector

**Supplementary Table 2.** Statistical significances between the focus-stacked and the best-focused images for each subject

**Supplementary Table 3.** Demographics and clinical characteristics of study subjects

**Supplementary Figure**

**Supplementary Figure 1.** The representative pair of focus-stacked and best-focused image in the inferior sector of the subject “14,” in which the focus-stacked image was not significantly superior in the informativeness to diagnose angle pathology and in the energy of Laplacian to the best-focused image.

**Supplementary Tables**

| **Supplementary Table 1. Statistical significances between the focus-stacked and the best-focused images for each sector** | | | | | | | | | | | | | | | | |
| --- | --- | --- | --- | --- | --- | --- | --- | --- | --- | --- | --- | --- | --- | --- | --- | --- |
| **Assessment** | **Sector** | | | | | | | | | | | | | | | |
|  | **ITT** | **IT** | **IIT** | **I** | **IIN** | **IN** | **INN** | **N** | **SNN** | **SN** | **SSN** | **S** | **SST** | **ST** | **STT** | **T** |
| Deepness of DOF (*P* value^*^) | <0.01 | <0.01 | <0.01 | <0.01 | <0.01 | <0.01 | <0.01 | <0.01 | <0.01 | <0.01 | <0.01 | <0.01 | <0.01 | <0.01 | <0.01 | <0.01 |
| Informativeness to diagnose angle pathology (*P* value^*^) | <0.01 | 0.02 | <0.01 | <0.01 | 0.02 | <0.01 | 0.08 | 0.08 | <0.01 | 0.02 | 0.08 | 0.02 | <0.01 | <0.01 | <0.01 | <0.01 |
| Energy of Laplacian (*P* value^*^) | <0.01 | <0.01 | <0.01 | <0.01 | <0.01 | <0.01 | <0.01 | <0.01 | <0.01 | <0.01 | <0.01 | 0.02 | <0.01 | 0.02 | <0.01 | <0.01 |

Abbreviations: DOF, depth of focus; ITT, inferior-temporal-temporal; IT, inferior-temporal; IIT, inferior-inferior-temporal; I, inferior; IIN, inferior-inferior-nasal; IN, inferior-nasal; INN, inferior-nasal-nasal; N, nasal; SNN, superior-nasal-nasal; SN, superior-nasal; SSN, superior-superior-nasal; S, superior; SST, superior-superior-temporal; ST, superior-temporal; STT, superior-temporal-temporal; T, temporal.

^*^Sign test.

| **Supplementary Table 2. Statistical significances between the focus-stacked and the best-focused images for each subject** | | | | | | | | | | | | | | | | |
| --- | --- | --- | --- | --- | --- | --- | --- | --- | --- | --- | --- | --- | --- | --- | --- | --- |
| **Assessment** | **Subject** | | | | | | | | | | | | | | | |
|  | **1** | **2** | **3** | **4** | **5** | **6** | **7** | **8** | **9** | **10** | **11** | **12** | **13** | **14** | **15** | **16** |
| Deepness of DOF (*P* value^*^) | <0.01 | <0.01 | <0.01 | <0.01 | <0.01 | <0.01 | <0.01 | <0.01 | <0.01 | <0.01 | <0.01 | <0.01 | <0.01 | <0.01 | <0.01 | <0.01 |
| Informativeness to diagnose angle pathology (*P* value^*^) | <0.01 | <0.01 | <0.01 | <0.01 | 0.21 | <0.01 | <0.01 | <0.01 | <0.01 | 0.02 | <0.01 | 0.21 | 0.08 | 0.80 | 0.45 | <0.01 |
| Energy of Laplacian (*P* value^*^) | <0.01 | <0.01 | <0.01 | 0.08 | <0.01 | <0.01 | <0.01 | <0.01 | <0.01 | <0.01 | <0.01 | <0.01 | <0.01 | 0.08 | <0.01 | <0.01 |

Abbreviations: DOF, depth of focus.

^*^Sign test.

| **Supplementary Table 3. Demographics and clinical characterictics of the study subjects** | | | | | | | |
| --- | --- | --- | --- | --- | --- | --- | --- |
| **Subject No.** | **Sex** | **Age** | **Diagnosis** | **Surgical history** | **Shaffer grade of angle width** | **Lens status** | **History of blunt injury** |
| 1 | M | 26 | JOAG | - | 4 | Phakic | - |
| 2 | M | 81 | Normal | - | 4 | Phakic | - |
| 3 | M | 70 | Normal | - | 4 | Phakic | - |
| 4 | F | 72 | SOAG (traumatic glaucoma) | - | 4 | Phakic | + |
| 5 | M | 83 | POAG | - | 4 | Phakic | - |
| 6 | M | 55 | SACG | LOT ab externo→Phaco+AGV into AC | 0 | IOL | - |
| 7 | F | 75 | PEG | Phaco+LOT ab externo→ExPress→AGV into AC | 4 | IOL | - |
| 8 | M | 66 | PEG | LEC→Phaco+LOT ab interno | 4 | IOL | - |
| 9 | F | 74 | PEG with PAS | Phaco+GSL | 3 | IOL | - |
| 10 | M | 71 | POAG | Phaco+LOT ab externo→AGV into AC→PPV+ERM peeling+AGV via pars plana | 4 | IOL | - |
| 11 | M | 67 | PEG | Phaco+LOT ab externo→ExPress | 4 | IOL | - |
| 12 | M | 76 | POAG | Phaco+ExPress→LEC | 4 | IOL | - |
| 13 | M | 50 | SOAG (uveitic glaucoma) | LEC→Phaco+PPV+AGV via pars plana | 3 | IOL | - |
| 14 | M | 76 | POAG | Phaco | 4 | IOL | + |
| 15 | M | 56 | SOAG (pigment dispersion syndrome) | Phaco+PPV→PPV→PPV＋IOL fixation | 4 | IOL | - |
| 16 | F | 68 | POAG | Phaco+iStent | 4 | IOL | - |

Abbreviations: No, number; JOAG, Juvenile-onset open-angle glaucoma; SOAG, secondary open-angle glaucoma; POAG, primary open-angle glaucoma; SACG, secondary angle closure glaucoma; PEG, pseudoexfoliation glaucoma; PAS, peripheral anterior synechiae; LOT, trabeculotomy; AGV, Ahmed glaucoma valve implantation; AC, anterior chamber; LEC, trabeculectomy; GSL, goniosynechialysis; PPV, pars plana vitrectomy; IOL, intraocular lens.

**Supplementary Figure**


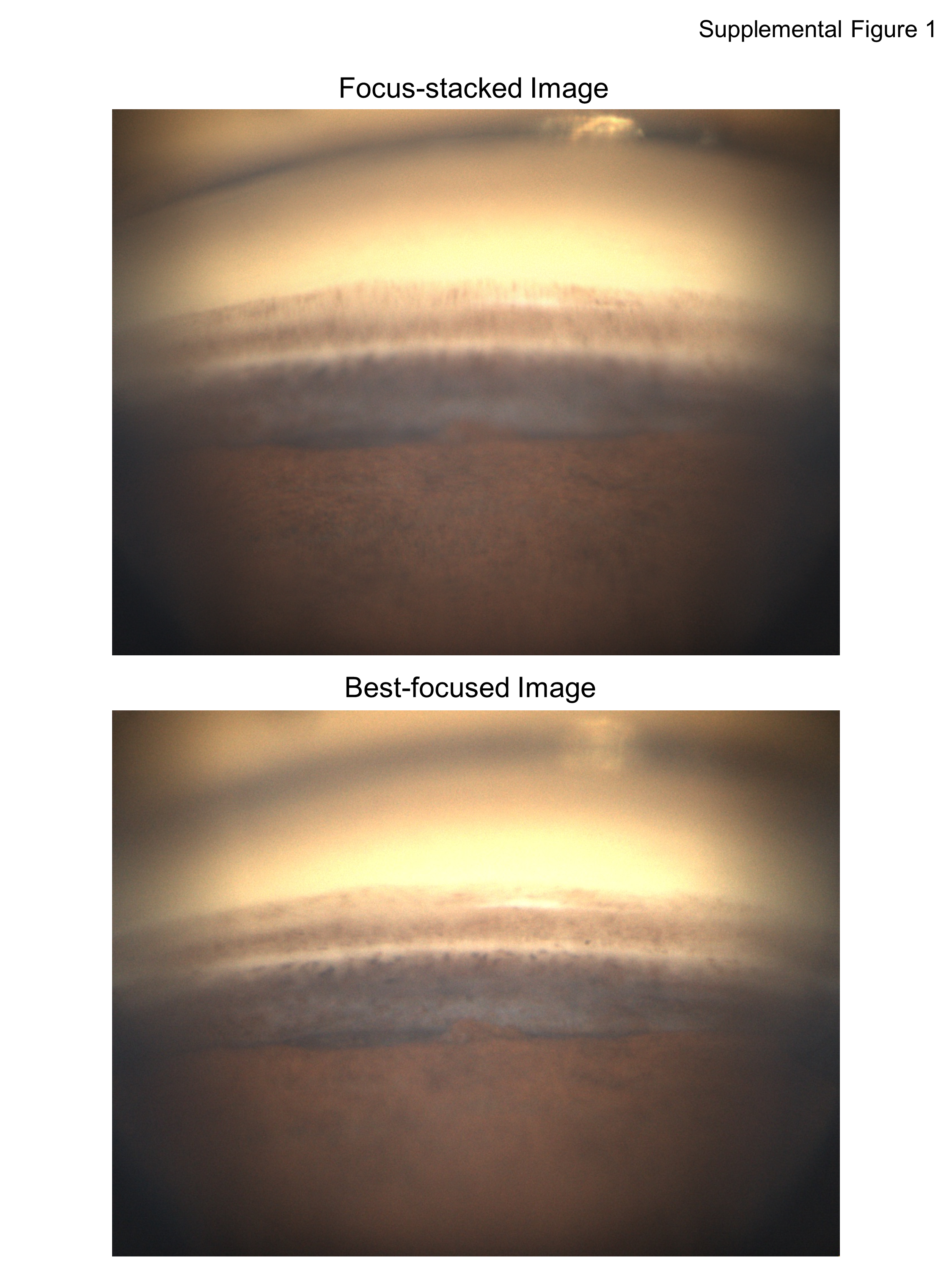


**Supplementary Figure 1.** The representative pair of focus-stacked and best-focused image in the inferior sector of the subject “14,” in which the focus-stacked image was not significantly superior in the informativeness to diagnose angle pathology and in the energy of Laplacian to the best-focused image. The images demonstrated the angle recession with an extreme wide-angle due to the widening of the ciliary body band. Thus, the captured best-focus image exhibited a wide range of in-focused areas without the image processing.
